# Efficacy of electrical stimulation combined with ultrasound acupuncture therapy on treatment in patients with intrauterine adhesions: Study protocol for a placebo-controlled, single-blind, single-center, randomized trial

**DOI:** 10.1097/MD.0000000000031469

**Published:** 2022-12-16

**Authors:** Tao Xu, Li Xie, Xiaoling Qin, Xueting Xie, Sien Mo, Qiu Jiang, Feng Liang, Xuehong Zhu, Bin Li, Zhong Lin

**Affiliations:** a Global Health Institute, School of Public Health, Xi’an Jiaotong University Health Science Center, Xi’an, Shaanxi, China; b Medical Affairs, Nanjing Midlander Medical Research Center, Nanjing, Jiangsu, China; c Clinical Research Institute, Shanghai Jiao Tong University School of Medicine, Shanghai, China; d The Reproductive Hospital of Guangxi Zhuang Autonomous Region, Nanning, China

**Keywords:** electrical stimulation, intrauterine adhesions, study protocol, ultrasound acupuncture

## Abstract

Recurrence of postoperative adhesions is one of the most important factors for poor reproductive outcomes after hysteroscopic surgery, particularly in cases diagnosed with severe intrauterine adhesions (IUAs), where the recurrence rate is significantly higher. This study aims to explore the effectiveness of the electrical muscle stimulation combined with ultrasound acupuncture therapy in preventing the recurrence of IUAs and improving reproductive outcomes after operative hysteroscopy.

**Methods:**

This study is a single-center, randomized controlled trial. A total of 210 patients with IUAs will be randomly assigned into 2 groups according to the ratio of 1:1, as the treatment group and the control group. Participants will receive the electrical muscle stimulation combined with ultrasound acupuncture therapy and oral hormone supplementation or receive oral hormone supplementation only. The primary outcome was the clinical response rate. There were 3 menstrual cycles of treatment and 3 menstrual cycles of follow-up in this study.

**Ethics and Dissemination:**

This study protocol was approved by the Ethics Committee of the Reproductive Hospital of Guangxi Zhuang Autonomous Region (approval number: KY-LL-2022-06). This trial will be conducted in accordance with the principles of the Declaration of Helsinki as well as Good Clinical Practice. Study results will be disseminated at academic presentations and publications in peer-reviewed journals.

**Trial Registration:**

Registry name: Clinical value of electroultrasonic instrument in the treatment of IUAs and changes of related protein expression; Registry number: ChiCTR2200058901; registration date: April 19^th^, 2022; http://www.chictr.org.cn/showproj.aspx?proj=166155.

**Strengths and limitations of this study**

• Observational studies have evaluated the effectiveness of prevention of recurrence of IUAs before, but the quality of evidence on the effectiveness of the ULTRA-EMS therapy device in improving reproductive outcomes of IUAs is still unsatisfactory.
• This study will evaluate the effectiveness of the ULTRA-EMS therapy device in preventing the recurrence of IUAs after operative hysteroscopy. It will improve the quality of the evidence on the effectiveness of the ULTRA-EMS therapy device preventing the recurrence of IUAs and improving reproductive outcomes after operative hysteroscopy.
• Recurrence of postoperative adhesions is one of the most important factors for poor reproductive outcomes after operative hysteroscopy. This therapy will help decrease the recurrence of IUAs and improve reproductive outcomes.

## 1. Introduction

Intrauterine adhesions (IUAs) were first reported by Fritsch in 1894.^[1]^ In 1950, Asherman systematically and comprehensively reported IUA through a summary of 29 IUA cases, so that people began to pay attention to and deeply studied this disease. Also, the IUAs is also known as Asherman syndrome.^[2]^ It is characterized by varying degrees of scar formation in the uterine cavity and is also the cause of menstrual disorders, infertility, and placental abnormalities.^[3,4]^ IUA occurs most often after infection or trauma, such as uterine surgery, especially in the postpartum period of cesarean section.^[5,6]^ A large number of inflammatory chemokines, together with cytokines and cell adhesion molecules, are aberrantly expressed during the development of IUA.^[7]^ The current report found that IUA was associated with decline in reproductive clinical outcomes after natural conception and assisted reproductive technologies.^[8]^ The prevalence of IUAs is currently not entirely consistent, ranging from 0.3% (an incidental finding in women with intrauterine device placement without gynecological symptoms) to 21.5% (women with postpartum curettage).^[9]^ Of all the tools used to diagnose IUA or evaluate the severity of IUA, hysteroscopy is the current gold.^[10]^ In the treatment, the recovery of uterine shape and function is mainly helped by surgical separation of the adherent uterine cavity, supplemented by physical isolation and hormone to promote endometrial regeneration.^[8]^ Operative hysteroscopy is also considered a good treatment for patients.^[11]^ The postoperative effectiveness of hysteroscopic separation of IUAs is generally satisfactory, and the probability of usual or improved menstruation after surgery can reach 92% to 96%.^[12]^ Recurrence of postoperative adhesions is one of the most important factors for poor reproductive outcome after operative hysteroscopy, especially in cases diagnosed with severe AS, where the recurrence rate is significantly higher.^[13]^ More and more scholars have focused on exploring the measures to prevent adhesion recurrence and improve postoperative pregnancy rate during and after operative hysteroscopy.^[10]^

Electrical stimulation, including Neuromuscular Electrical Stimulation (NMES) and transcutaneous electrical nerve stimulation (TENS), has been applied in the treatment of stress urinary incontinence, pelvic pain, and sexual dysfunction.^[14]^ Some researchers also did a preliminary study of NMES in reproductive medicine and have improved the clinical pregnancy rate.^[15]^ The mechanism of action in NMES and TENS is related to the mechanism of recurrence of IUAs. Acupuncture has many applications in treating various reproductive disorders, such as polycystic ovary syndrome, pain induced by oocyte retrieval, diminished ovarian reserve, embryo transfer, and oligospermia/asthenospermia.^[16–18]^ Some observational studies have evaluated the effectiveness of prevention of recurrence of IUAs.^[19]^ The electrical muscle stimulation combined with ultrasound acupuncture (ULTRA-EMS) therapy device C7000 (Nanjing Medlander Medical Technology Co. Ltd, Nanjing, China), combined TENS, NMES, and ultrasound acupuncture and has been used for reproductive medicine. Nevertheless, this combination has not been evaluated for the treatment or prevention of recurrence of IUAs after operative hysteroscopy.

This study aims to evaluate the effectiveness of ULTRA-EMS in preventing the recurrence of IUAs after operative hysteroscopy.

## 2. Methods

### 2.1. Objectives

This trial is aimed to evaluate the effectiveness of the ULTRA-EMS therapy device in preventing the recurrence of IUAs and improving reproductive outcomes after operative hysteroscopy.

### 2.2. Study design

This is a randomized, single-center clinical trial which aimed to evaluate the effectiveness of electrical stimulation combined with ultrasound acupuncture to treat IUAs. This randomized trial was conducted in one single center: Guangxi reproductive hospital. A total of 210 patients diagnosed with IUAs were enrolled in this trial.

### 2.3. Participants

**Figure 1.**
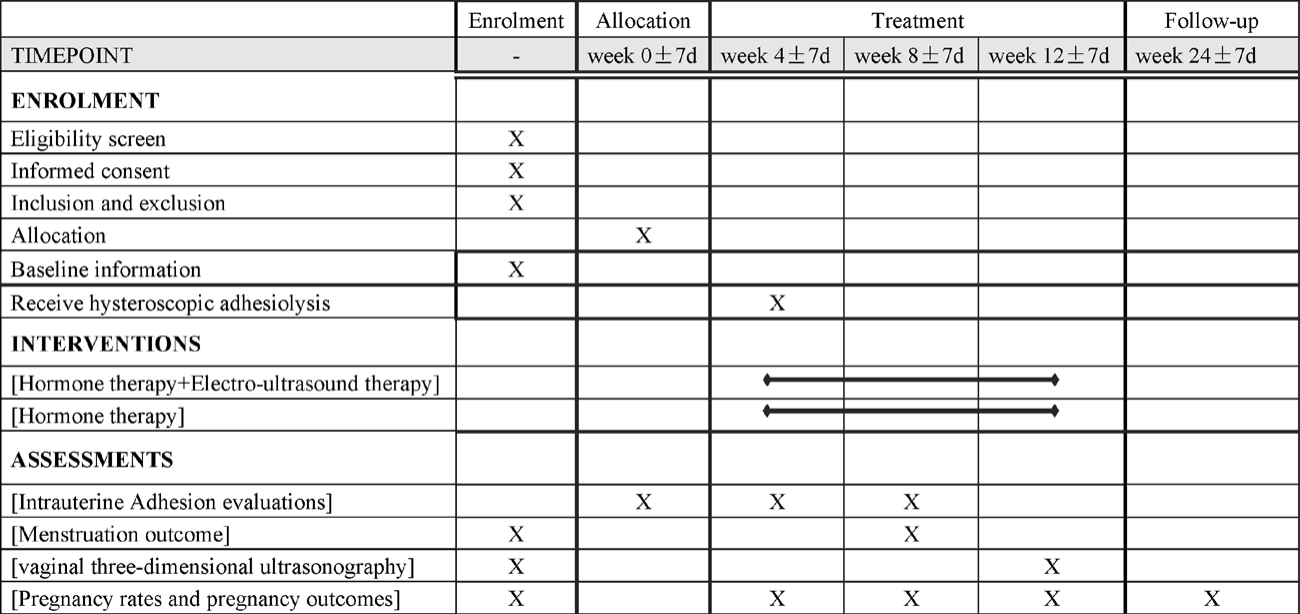
Schedule of enrolment, interventions, and assessments.

Patients were recruited only if they met all inclusion criteria and did not meet the exclusion criteria. Figure 1 is flow diagram of enrollment of study participants.

Inclusion criteria:

(1) Female aged between 20 and 40 years;
(2) According to practice guidelines on IUAs developed in collaboration with the European Society of gynecological endoscopy (esge), hysteroscopy is the most accurate method for diagnosis of IUAS,^[12]^ and we diagnosed the IUAS with hysteroscopy;
(3) Patients were willing to accept hysteroscopic adhesiolysis;
(4) Regular menstrual cycle;
(5) Sign informed consent.

Exclusion criteria:

(1) Acute and subacute genital inflammation and pelvic infection;
(2) Uterine perforation repair within 6 months;
(3) Cervical cancer, adenomyosis, uterine fibroids (nonsubmucosal) > 4 cm, endometrial cancer, and other uterine tumors;
(4) Genital tract tuberculosis, without antituberculosis treatment;
(5) Suffering from serious diseases: such as heart, lung, liver, kidney and other medical diseases, coagulation dysfunction and bleeding disorders, difficulty tolerating surgery;
(6) Taking hormone drugs in the past 1 month;
(7) Combined with endocrine-related diseases (such as ovarian dysfunction, thyroid disease);
(8) Patients with moderate and severe IUAs after 2 or more intrauterine adhesiolysis.

### 2.4. Randomization and participants allocation

Block randomization was used in this study with a block size of 4. A randomization table (containing randomization information including random seeds, block length, and number) was generated by the statistical professional of the Clinical Research Institute of Shanghai Jiao Tong University School of Medicine based on the block randomization method. The randomization table will be sealed in duplicate and will be kept by the primary research institution undertaking the clinical trial (the filled-in randomization table shall not be opened before the end of the question). At the same time, a corresponding randomization letter was prepared for each subject. The envelope will be labeled with the subject number. The sealed envelope indicated the assigned treatment for the issue, and the statistical unit was responsible for sending the randomization letter to the study site. The study site must open the sealed stationery in the order of the cases and dispose of them according to the randomly assigned group in the paper and the corresponding treatment. The opening date and time should be recorded and signed by the sponsor.

### 2.5. Interventions

There were 2 phases in this study. Once the patients finished hysteroscopic adhesiolysis, they would accept treatment according to the dividing of each group for 2 menstrual cycles. After the first phase of treatment, operative hysteroscopy would be carried out for all of these patients to evaluate restoring the uterine cavity. Patients will be treated for one menstrual cycle during the second phase, depending on the group. Moreover, we then conducted a logical image examination of other outcomes after that.

#### 2.5.1. Treatment group.

The patients in the treatment group will receive electrical stimulation combined with ultrasound acupuncture and oral hormone supplementation for 3 menstrual cycles. Treatment would begin on the day the patient underwent hysteroscopic adhesiolysis. Estrogen progestogen sequential therapy is as follows: during the menstrual cycle of hysteroscopic adhesiolysis, on day 2 after hysteroscopic adhesion release, oral estradiol valerate 2 mg (Progynova; Schering, Weesp, The Netherlands) was administered twice daily for 21 days; from day 10, oral dydrogesterone 10 mg twice daily for 10 days. Those with menstruation began to take the next cycle on the fifth day of menstruation, and those without menstruation began to take the next cycle after 7 days of drug withdrawal. Three consecutive menstrual cycles would be taken.

Electrical stimulation combined with ultrasound acupuncture therapy is as follows: the device is called ULTRA-EMS THERAPY DEVICE MLD C7000 (Nanjing Medlander Medical Technology Co. Ltd, Nanjing, China). During the menstrual cycle, Patients would be treated with TENS and ultrasound acupuncture modalities for 7 days from the first day after the patient undergoes hysteroscopic adhesion release. Each patient visit lasted approximately 30 minutes. TENS includes 8 electrode pads, which correspond to different body positions, as shown in the figure below. Table 1 shows the TNES and acupuncture points. The electrode piece used for TENS is a body surface electrode-circular 50 mm (viscose electrode piece), which is reusable and specially used by specially assigned persons. It is discarded after ten times of use. Stimulation current intensity is as follows:: adjustable within the range of 0 to 100 mA, adjustable with the step of 0.5 mA. Intensity adjustment is based on the patient’s tolerance to the degree that the patient can feel the current stimulation. When the patients accept TENS, the doctors put electrode pieces on the surface of the body (S1-8). The ultrasound acupuncture includes a therapeutic ultrasound probe and ultrasonic therapy fixing paste. Therapy fixing paste is used for applying an ultrasonic probe to acupoints on the body surface (A1-8). The ultrasound acupuncture mode has 10 shifts with adjustable and effective sound intensity: less than 3 W/cm^2^. Typically, we used the sixth shift for patients. During the 2 to 3 menstrual cycle, TENS, ultrasound acupuncture, and intermittent vaginal electrical stimulation would be used for patients from 5 to 7 days of the menstrual cycle. TENS and ultrasound acupuncture are the same as the first menstrual cycle. Intermittent vaginal electrical stimulation is as follows: all women had their own vaginal probe (Nanjing Medlander Medical Technology Co. Ltd, Nanjing, China, MLD CV01). Doctors applied ointment (conductive gel) to the probe tip prior to insertion into the vagina and inserted the stylet approximately 6 to 8 cm deep. An adhesive electrode will be placed on the skin outside the vagina (on the female thigh). The vaginal probe will be left in the vagina, and electrical stimulation was started. The selected parameters included a biphasic intermittent biphasic current with a frequency of 40 Hz, a pulse width of 250 us, and a current intensity between 0 and 120 mA. On-off (duty cycle) periods were individually adjusted based on each female’s ability to maintain voluntary contractions. TENS, ultrasound acupuncture, and intermittent vaginal electrical stimulation would last about 30 minutes every time.

**Table 1:** Description of transcutaneous electrical nerve stimulation and acupuncture points.

**Stimulation type**	**Name**	**Localization**	**Point**
TENS	S1	Left external iliac artery, left groin	NA
TENS	S2	Right external iliac artery, right groin	NA
TENS	S3	Left femoral surface projection area, left gastrocnemius surface projection area	NA
TENS	S4	Right femoral surface projection area, right gastrocnemius surface projection area	NA
TENS	S5	Pelvic surface projection area, dorsal penetration (upper left)	NA
TENS	S6	Pelvic surface projection area, dorsal penetration (left lower)	NA
TENS	S7	Pelvic surface projection area, dorsal penetration (upper right)	NA
TENS	S8	Pelvic surface projection area, dorsal penetration (right lower)	NA
Ultrasound acupuncture	A1(left), a2(right)	In the flat umbilicus, 2 inches from the umbilicus, the width of the navel to the left and right 3 fingers	Tianshu
Ultrasound acupuncture	A3(left), a4(right)	The patient was placed in the decubitus position, 4 inches below the umbilicus (5 finger widths) and 3 inches aside (4 finger widths)	Zigong
Ultrasound acupuncture	B1(left), b2(right)	Calf nose (sitting or supine position, when the lower limbs are strongly pushed straight, located in the depression below the rubber cover, generally known as “external knee eye”) 3 inches below (4 finger width)	Zusanli
Ultrasound acupuncture	B3(left), b4(right)	Located on the medial side of the lower leg, 3 inches above the medial malleolus (4 finger width)	Sanyinjiao

NA= not available, TENS = transcutaneous electrical nerve stimulation.

#### 2.5.2. Control group.

The control group received oral hormone supplementation for 3 menstrual cycles. The protocol was the same with oral hormone supplementation in the treatment group.

### 2.6. Outcome measures

#### 2.6.1. Primary outcome.

a) Improvement of IUAs

The primary outcome of our study was the clinical response rate. After 2 menstrual cycles of treatment (about 2 months), hysteroscopy was performed to evaluate the recovery of endometrial cavity function. According to the recovery status, the clinical outcomes were classified into 3 levels (cure, improvement, and failure). These 3 stages had different standards. The first is the cure: after intrauterine treatment, the size and shape of the uterine cavity were normal, and the uterine angle and tubal opening were normal. The second is the improvement: IUAs score decreased after treatment. The severity of adhesions according to the American Fertility Society (AFS) IUA 19 classification.^[20]^ The last is the failure: they were unchanged clinical symptoms and a massive IUA condition. Overall response rate = (cure + improvement)/total number of cases *100%.

b)IUA evaluations

Before treatment, we evaluated and scored the severity of adhesions according to the American Fertility Society (AFS) IUA 19 classification.^[20]^ One of 2 reproductive surgeons in our hospital performed all operative hysteroscopies using the same techniques. During the operative hysteroscopy, the doctors then did hysteroscopic adhesiolysis for patients. Scores of 1 to 4 represent mild adhesions, 5 to 8 points for moderate adhesions, and 9 to 12 points for severe adhesions. Table 2 shows the details of the evaluation of IUA.

**Table 2:** Score assessment of intrauterine adhesion.

**Evaluation variable**	**Score**
**Extent of cavity involved**	
<1/3	1
1/3–2/3	2
>2/3	4
**Type of adhesions**	
Filmy	1
Filmy + dense	2
Dens	4
**Menstrual pattern**	
Normal	0
Hypomenorrhea	2
Amenorrhea	4

#### 2.6..2. Secondary outcomes.

a)Menstruation outcome

We assessed the amount of menstrual status according to Higham’s pictorial blood loss assessment chart.^[21]^ Methods: 240 mm sanitary napkins were uniformly used, and the blood penetration area of each piece of switched sanitary napkins was recorded. Mild: blood penetration area ≤ 1/3 of 1 point of the total area of the sanitary napkin. Moderate: the blood penetration area accounts for 1/3 to 3/5 of the 5 points of the entire sanitary napkin area. Severe: the bleeding area is basically 20 points of the entire sanitary napkin area. The size of the lost blood clot is 1 point for small blood clots < 1 coin; 5 points for large blood clots ≥ 1 coin. When blood clots could not represent blood loss, the percentage of the recorded blood volume was estimated for recording. The score, quantity and days of each sanitary napkin were filled in the sanitary napkin count and score table. Table 3 shows the menstrual blood loss record card. Menstrual blood loss score = (number of sanitary napkins with a large amount of blood-stained × 20) + (number of sanitary napkins with a medium amount of blood-stained × 5) + (number of sanitary napkins with a small amount of blood-stained × 1) + (number of blood clots < 1 coin × 1) + (blood clots ≥ 1 coin × 5). We recorded the menstrual status before and after 2 menstrual cycles of treatment.

**Table 3:** Record card for menstrual blood loss and assessment time point.

	**Menstrual cycle**						
**Blood staining level of sanitary napkin**	**1st day**	**2nd day**	**3rd day**	**4th day**	**5th day**	**6th day**	**7th day**
Severe							
Moderate							
Mild							
Blood clots < 1 coin							
Blood clots ≥ 1 coin							
Menstrual color							
Dysmenorrhea							

b)*Changes in 3-dimensional vaginal ultrasonography*

The changes in endometrial structure and hemodynamics before and after treatment will be evaluated in both groups. Vaginal 3-dimensional ultrasonography was performed by the same physician (attending and above grade) during ovulation before treatment (12 to 14 days before menstruation) and ovulation after the third course of treatment, mainly measuring endometrial type, endometrial thickness (ED), endometrial volume, endometrial classification (A, B, C), uterine artery blood flow (PI, RI, S/D, PS), endometrial vascular index (VI), blood flow index (FI), vascular blood flow index (VFI), and blood flow classification (I, II, and III).

c) *Pregnancy rates and pregnancy outcomes*

Pregnancy rate and pregnancy outcome would be follow-up at 2 and 6 months after operation in both groups. The purpose of the follow-up was to evaluate the long-term effectiveness of the electro-ultrasound device intreating IUAs. Researchers will pay close attention to the patient’s pregnancy during the follow-up period.

### 2.7. Withdrawn

The reason for participants who withdrew would be recorded for the withdrawn (e.g., adverse event, lack of effectiveness).

### 2.8. Study timeline

**Figure 2.**
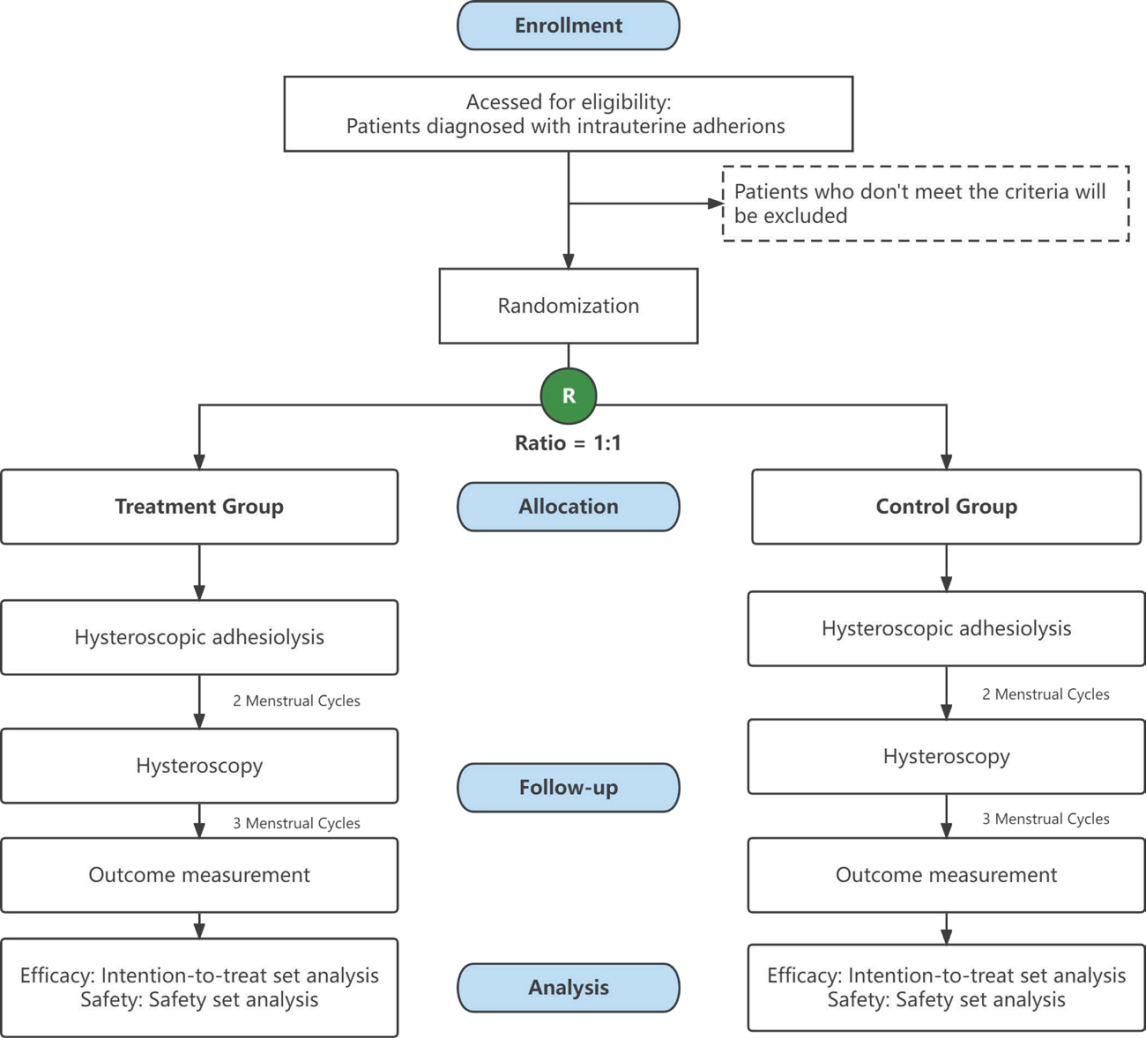
Flow diagram of enrollment of study participants.

The study is expected to take 12 months to complete data collection, including 7 times of data collection. According to SPIRIT,^[22]^ the schedule of interventions and assessments is shown in Figure 1. The flow chart of enrollment of study participants was shown in Figure 2.

### 2.9. Statistical considerations

#### 2.9.1. Sample size estimation.

The number of patients required for this trial was determined on the basis of the primary outcome of the clinical response rate. With an estimated clinical response rate of 56.6% in the control group, we calculated that 168 participants would be required for the trial to achieve 80% power (β = 0.2) to detect a 20% difference in a clinical response rate increase in the trial groups. The allocation ratio was 1:1, at a 2-sided overall significance level of 5% (α = 0.05). The test statistic used was the 2-sided Z-Test with unpooled variance. With an estimated follow-up loss of 20%, we aimed to recruit 105 participants per group, which was 210 participants in total.

#### 2.9.2. Statistical analysis plan.

A descriptive analysis of the baseline characteristics of the participants will be performed. Continuous variables will be presented as means and standard deviations; categorical variables will be presented as medians and interquartile ranges (lower and upper quartiles). The Kolmogorov–Smirnov test and histogram analyses will used to determine whether continuous variables are normally distributed. Levene’s test will used for the evaluation of homogeneity of variances. Two independent groups of parametric variables will be compared using a Student’s *t* test. For non-parametric variables, the Mann–Whitney *U* test will administered.

The principles of intention-to-treat analysis will be used. The primary analysis dataset was the intention-to-treat set. Both primary and secondary outcomes will be compared between the trial and control groups for the superiority of the trial group over the control group. For primary outcome comparisons of clinical efficacy, categorical data were analyzed by Chi-square or Fisher’s exact test, as appropriate. All trial results will be presented as a summary of the outcome measures, together with the estimated effect size and its confidence interval. All data analysis will be performed by using the IBM SPSS 26. The significance level are established at 2 sides 0.05, and the confidence interval limits at 95%.

### 2.10. Interim analysis and monitoring

No interim analysis has been planned.

### 2.11. Missing values

In principle, the missing values are not supplemented.

### 2.12. Data entry and quality control of data

Data collection will begin once study participants are informed and enrolled. Data entry will be primarily electronic. To preserve confidentiality, data will be stored on secure servers and the authors had no access to information that could identify individual participants during or after data collection. All data analysis will be conducted with condition masked.

## 3. Discussion

This trial was a randomized, single-center clinical trial and aimed to evaluate the effectiveness of the ULTRA-EMS therapy device in the prevention of recurrence of IUAs. Endometrial damage is a direct factor in the formation of IUA, mainly due to tissue basal layer injury, promoting neovascularization. In the process, uterine tissue secreted a large number of inflammatory factors, leading to the occurrence of endometrial fibrosis.^[23]^

Electrical stimulation accelerated blood flow, maintained reduced blood flow resistance, increased blood supply, and promoted endometrial growth by controlling the contraction and relaxation of vascular smooth muscle, which might be the potential mechanism. Acupuncture and moxibustion had the effects of dredging meridians, adjusting viscera and immune function, and had a better promoting effect on embryo implantation. Acupuncture and moxibustion could effectively increase the level of molecular markers of endometrial tissue by regulating the reproductive axis of “HPO,” thereby improving endometrial receptivity.^[16]^ We used focused ultrasound to stimulate acupoints to simulate acupuncture, which had the advantages of easy operation and painlessness compared with traditional acupuncture.

## Acknowledgments

We are very grateful to the participating women. We would also like to thank all the clinical researchers and research nurses at the participating centers for their efforts. We would like to thank Dr Ruijia Yang, chief president of Nanjing Midlander Medical Research Center, for his help in revising the manuscript.

## Author contributions

XX researched the literature, and ZL, TX, XX, and LX conceived the study. TX, LX, XX, XQ, SM, and QJ were involved in protocol development, gaining ethical approval, patient recruitment and data analysis. TX and LX drafted the manuscript. The logistical aspects of the start of the trial were the responsible by FJ, XJ, and LB. All the authors read and approved the final manuscript.

**Conceptualization:** Xu Tao, Li Xie, Zhong Lin.

**Data curation:** Xu Tao.

**Funding acquisition:** Zhong Lin.

**Investigation:** Xiaoling Qin, Qiu Jiang, Feng Liang, Xuehong Zhu, Bin Li.

**Methodology:** Xu Tao, Li Xie, Xiaoling Qin, Zhong Lin.

**Project administration:** Zhong Lin.

**Software:** Xueting Xie.

**Writing – original draft:** Xu Tao, Li Xie, Zhong Lin.

**Writing – review & editing:** Xiaoling Qin, Xueting Xie, Qiu Jiang, Feng Liang, Xuehong Zhu, Bin Li, Zhong Lin.

**Writing – review & editing:** Xu Tao, Li Xie.
